# Multilineage Lymphoblastic Lymphoma as an Initial Presentation of Mixed Phenotype Acute Leukemia

**DOI:** 10.1155/2023/3628712

**Published:** 2023-02-25

**Authors:** Mako Ikeda, Wataru Nakahara, Mizuki Asako, Yuka Umeki, Yoshiki Matsuoka, Takuya Terakawa, Hitomi Matsunaga, Yuki Iwasa, Riko Saito, Yuki Iwama, Takahiro Matsui, Kazumasa Oka, Shuji Ueda

**Affiliations:** ^1^Department of Hematology, Hyogo Prefectural Nishinomiya Hospital, Nishinomiya, Hyogo, Japan; ^2^Department of Clinical Laboratory, Hyogo Prefectural Nishinomiya Hospital, Nishinomiya, Hyogo, Japan; ^3^Department of Radiology, Hyogo Prefectural Nishinomiya Hospital, Nishinomiya, Hyogo, Japan; ^4^Department of Pathology, Osaka University Graduate School of Medicine, Suita, Osaka, Japan; ^5^Department of Pathology, Hyogo Prefectural Nishinomiya Hospital, Nishinomiya, Hyogo, Japan

## Abstract

Mixed phenotype acute leukemia (MPAL) is characterized by leukemic blasts that express markers of multiple lineages. Compared with acute myeloid leukemia (AML) and acute lymphoblastic leukemia (ALL), MPAL is considered to have a poor treatment outcome. We report a case of MPAL T/myeloid not otherwise specified that was initially presented as multilineage lymphoblastic lymphoma and subsequently developed into leukemic MPAL. An acute lymphoblastic leukemia-based treatment regimen was ineffective, but azacitidine and venetoclax therapy resulted in hematological complete remission. Our case suggests that multilineage lymphoblastic lymphoma should be considered to be the same disease as MPAL, albeit with different clinical presentations. Optimal treatment for MPAL has not been established yet, but azacitidine and venetoclax therapy may be a potential approach.

## 1. Introduction

Mixed phenotype acute leukemia (MPAL), which accounts for less than 5% of all acute leukemia, is a rare subtype of acute leukemia in which blast cells express markers of multiple developmental lineages [1]. According to the 2016 revision of the World Health Organization (WHO) classification, MPAL is classified as acute leukemia of ambiguous lineage [2]. Such leukemia is classified into five categories: Philadelphia chromosome-positive MPAL; MPAL with t(v:11q23.3); MPAL B/myeloid not otherwise specified (NOS); MPAL T/myeloid, NOS; and acute undifferentiated leukemia. Compared with acute myeloid leukemia (AML) and acute lymphoblastic leukemia (ALL), MPAL is considered to have a poor treatment outcome in terms of overall survival and the probability of achieving complete remission.

MPAL is diagnosed by confirming the presence of leukemic cells with extensive coexpression of myeloid and lymphoid markers in a single blast population (biphenotypic) or in two or more separate blast populations of distinct lineages (bilineal) [3]. On the other hand, unusual cases of MPAL with biphenotypic blasts in lymph nodes without medullary involvement have been also reported [4–6], and Martin-Guerrero et al. described these cases as “nonleukemic” MPAL [4].

Here, we present a case of T/Myeloid MPAL that was initially presented as bilineal lymphoblastic lymphoma and subsequently developed into leukemic MPAL. An ALL-based treatment regimen comprising doxorubicin, cyclophosphamide, vincristine, and prednisolone was ineffective, but complete hematological remission was achieved for 6 months with azacitidine and venetoclax therapy.

## 2. Case Presentation

**Figure 1:**
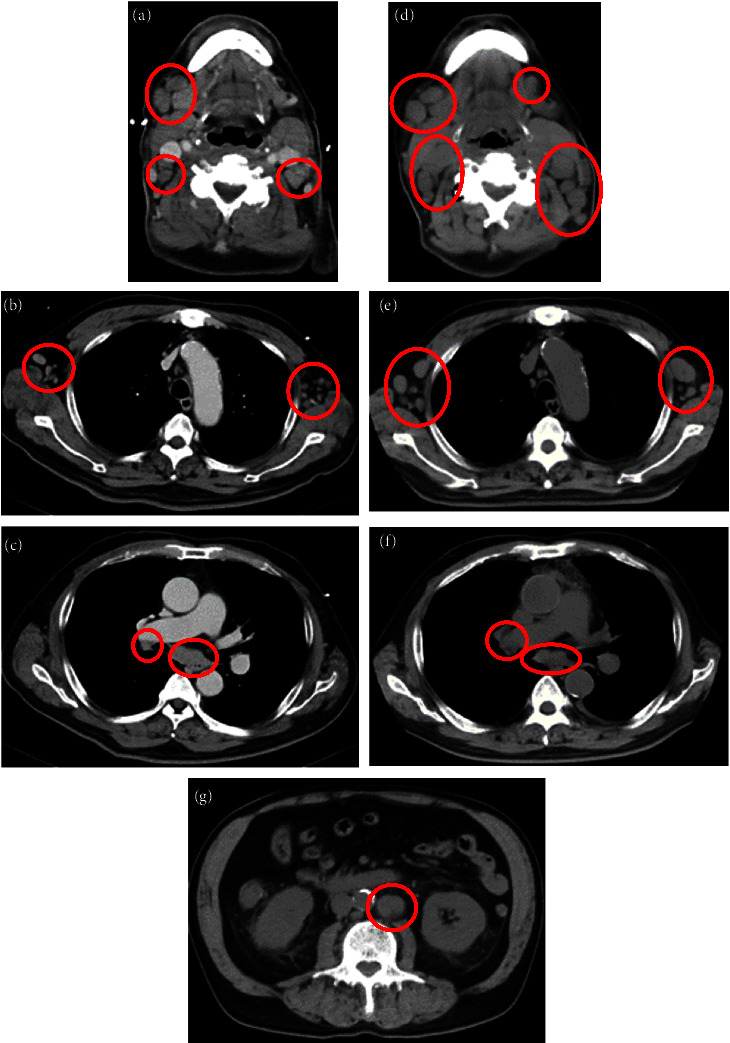
Computed tomography revealed multiple lymph node swellings. Computed tomography scans at the first visit (a, b, and c) and four months later (d, e, f, and g), shows the cervical lymph nodes (a, d), axillary lymph nodes (b, e), mediastinal lymph nodes (c, f), and para-aortic lymph node (g).

**Figure 2:**
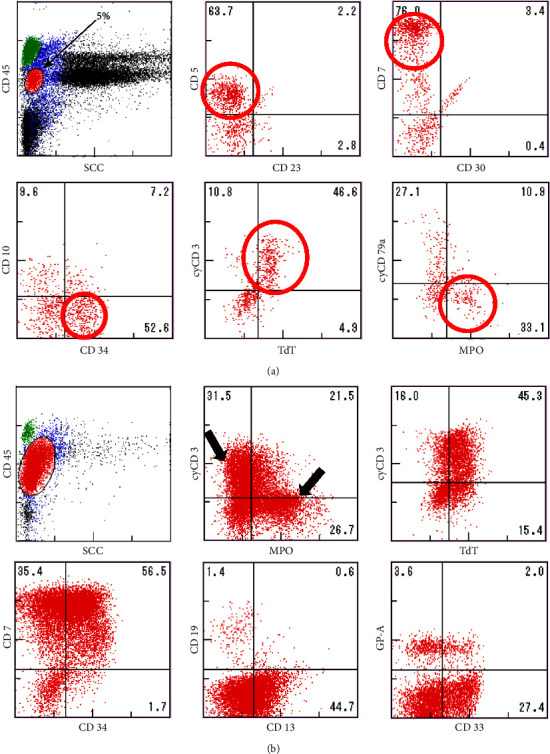
Flow cytometry analysis of bone marrow cells. (a) Flow cytometry showed a small immature cell population coexpressing T cell and myeloid markers. (b) Two populations of leukemic cells were found: one with myeloperoxidase (MPO) strongly positive and cytoplasmic CD3 (cyCD3) weakly positive cells and the other with MPO weakly positive and cyCD3 strongly positive cells. cyCD3, cytoplasmic CD3; MPO, myeloperoxidase; TdT, terminal deoxynucleotidyl transferase.

A 71-year-old man presented with bilateral cervical lymphadenopathy. Computed tomography revealed cervical, axillary, and mediastinal lymph node swelling measuring up to 20 mm (Figures 1(a) –1(c)). Initial laboratory findings, including complete blood count, complete metabolic panel, lactate dehydrogenase (LDH), and C-reactive protein (CRP), were normal, except for an increase in the soluble interleukin-2 receptor (sIL-2R) level (1048 *μ*/mL; reference value, <496 *μ*/mL). The May–Giemsa stain of the bone marrow aspirate did not detect any abnormal cells, but in flow cytometry, the CD45/side scatter (SCC) gating procedure identified a small immature cell population coexpressing T cell and myeloid markers, terminal deoxynucleotidyl transferase (TdT), cytoplasmic CD3, CD34, and myeloperoxidase (MPO) [7] (Figure 2(a)). Because the patient was asymptomatic and lymph node swelling was not increasing, the patient was followed up without treatment.

**Figure 3:**
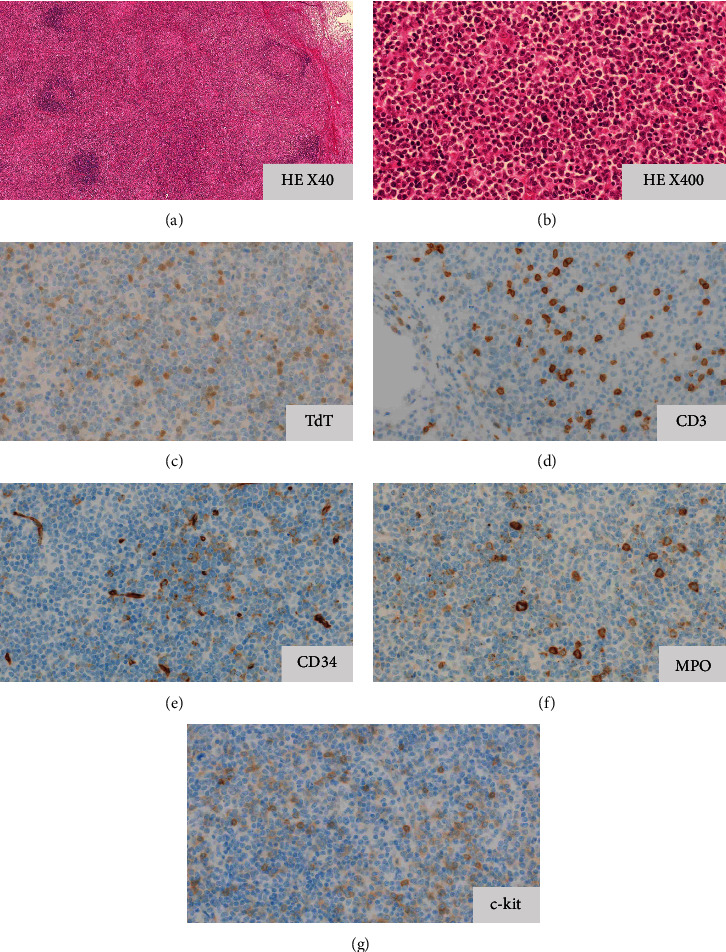
Histopathological examination of a cervical lymph node biopsy showing T/myeloid mixed phenotype acute leukemia cells, indicating extramedullary involvement. Hematoxylin and eosin stain ((a) x40; (b) x400) and immunohistochemical stain for terminal deoxynucleotidyl transferase (c), CD3 (d), CD34 (e), myeloperoxidase (f), and c-kit (g). HE, hematoxylin and eosin; MPO, myeloperoxidase; TdT, terminal deoxynucleotidyl transferase.

Four months after the first visit, the patient was referred to our hospital with a high fever (38.7°C). A peripheral blood count showed anemia and thrombocytopenia (white blood cell count, 3.7 × 10^9^/L; hemoglobin level, 94 g/L; and platelet count, 53 × 109/L). Morphologic analysis of peripheral blood showed 29.5% blast cells. Examination of the bone marrow aspirate by May–Giemsa staining confirmed that 94.8% of cells were blasts. The MPO expression of blasts was analyzed by two methods, cytochemical staining and flow cytometry; in cytochemical MPO staining, large blasts showed strong positive staining and small blasts showed weak positive staining. In flow cytometry analysis, the CD45/side scatter (SCC) gating procedure revealed two blast populations: MPO strongly positive and cytoplasmic CD3 (cyCD3) weakly positive blasts, and MPO weakly positive and cyCD3 strongly positive blasts (Figure 2(b)). Both populations were positive for CD7, TdT, and CD13 (Figure 2(b)). Chromosomal karyotyping revealed a normal karyotype (46 XY). Molecular analyses showed no MLL or BCR-ABL rearrangement. Biochemical analysis revealed impairment of renal function (creatinine, 152 *μ*mol/L) and elevation of LDH (524 IU/L), sIL-2R (4773 *μ*/mL), and CRP (234 mg/L). A computed tomography (CT) scan showed that the lymph nodes had further increased in size since the CT scan was performed four months previously, and para-aortic lymphadenopathy was also noted (Figures 1(d) –1(g)). A biopsy of the left cervical lymph node showed sparsely distributed lymphoid follicles with interfollicular infiltration of atypical cells, which had a fine blastoid chromatin pattern. These blastoid cells were positive for TdT, CD34, MPO, CD3, and c-kit (Figures 3(a)–3(f)).

On the basis of the findings, the patient was diagnosed with MPAL T/myeloid NOS according to the 2016 revision of the WHO classification. Induction chemotherapy was started with an ALL-based regimen consisting of doxorubicin, cyclophosphamide, vincristine, and prednisolone. However, this treatment was ineffective, and the disease progressed. Therefore, the patient was treated with azacitidine (75 mg/m^2^ intravenous infusion for 7 consecutive days) and venetoclax (100 mg oral administration on day 1, 200 mg on day 2, and 200 mg with fluconazole on days 3–21).

After the first cycle of the new treatment regimen, the percentage of blasts in the bone marrow decreased to 5.2%, and normal hematopoiesis was restored. Hematological complete remission (HCR), including disappearance of lymphadenopathy, was achieved after two cycles of azacitidine and venetoclax therapy. We continued this regimen as an outpatient treatment and maintained HCR for 6 months. Subsequently, the patient relapsed with an increase in bone marrow blasts and lymphadenopathy. Eight months after the start of azacitidine and venetoclax treatment, the patient died from the disease.

## 3. Discussion

Most cases of acute leukemia can be classified as either AML or ALL, and only a few cases are MPAL, a small heterogeneous group of acute leukemia with features of both AML and ALL [1]. ALL and lymphoblastic lymphoma (LBL) are both derived from immature lymphocytes and are considered to be the same disease with different clinical presentations. In LBL, the main lesion is in an extramedullary site, such as the lymph node, thymus gland, liver, or spleen, with no or minimal (<25%) evidence of peripheral blood or bone marrow involvement; a small amount of bone marrow infiltration is often observed in LBL [8].

At the initial presentation, in our case, the main lesion was lymphadenopathy, with very small amounts of tumor cells in the bone marrow confirmed by flow cytometry. A later lymph node biopsy showed collapse of the normal follicle architecture and infiltration of neoplastic cells with blastoid morphology. These neoplastic cells were positive for CD34, TdT, MPO, and c-kit, i.e., they showed the features of MPAL. Our patient initially presented with multilineage lymphoblastic lymphoma, which subsequently developed into leukemic MPAL. According to our research, several cases of LBL with features of MPAL have been reported [4–6]. Our case suggests that multilineage lymphoblastic lymphoma and MPAL should be considered to be the same disease with different clinical presentations, similar to the relationship between LBL and ALL.

In our patient, a regimen based on ALL treatment was not effective, but azacitidine and venetoclax therapy induced hematological remission. By continuing outpatient treatment, the patient was able to maintain remission for about 6 months with adequate quality of life. Treatment with azacitidine and venetoclax was shown to be more effective in cases of AML with p53 or FLT3 mutation that had a poor clinical response to conventional combination chemotherapy [9, 10]. Recently, studies have shown that venetoclax may also be effective for ALL [11–13]. In addition, several case reports describe the efficacy of venetoclax combined with hypomethylating agents such as azacitidine and decitabine in MPAL that did not respond to conventional chemotherapy [14–16]. However, only one case of T/myeloid MPAL has been reported, in which venetoclax combined with decitabine showed efficacy [16]. To the best of our knowledge, our report presents the first case of MPAL T/myeloid NOS, in which venetoclax and azacitidine therapy was effective in achieving remission. Currently, there is no consensus regarding the optimal treatment for MPAL, but azacitidine and venetoclax therapy may be a potential therapeutic option.

## Data Availability

The data used to support the findings of this study are included within the article.
